# Marriage Patterns and Childbearing: Results From a Quantitative Study in North of Iran

**DOI:** 10.5539/gjhs.v8n3p1

**Published:** 2015-09-21

**Authors:** Ziba Taghizadeh, Fereshteh Behmanesh, Abbas Ebadi

**Affiliations:** 1 Department of Midwifery and Reproductive Health, School of Nursing and Midwifery, Tehran University of Medical Sciences, Tehran, Iran; 2 Reproductive Health, School of Nursing and Midwifery, Tehran University of Medical Sciences, Tehran, Iran; 3 Behavioral Sciences Research Center and Nursing Faculty of Baqiyatallah University of Medical Sciences, Tehran, Iran

**Keywords:** traditional marriage, modern marriage, intergrated marriage, marriage patterns, reproductive behaviors, childbearing

## Abstract

Social changes have rapidly removed arranged marriages and it seems the change in marriage pattern has played a role in childbearing. On the other hand, there is a great reduction in population in many countries which requires a comprehensive policy to manage the considerable drop in population. To achieve this goal, initially, the factors affecting fertility must be precisely identified. This study aims to examine the role of marriage patterns in childbearing. In this cross-sectional quantitative study, 880 married women 15-49 years old, living in the north of Iran were studied using a cluster sampling strategy. The results showed that there are no significant differences in reproductive behaviors of three patterns of marriage in Bobol city of Iran. It seems there is a convergence in childbearing due to the different patterns of marriage and Policymakers should pay attention to other determinants of reproductive behaviors in demographic planning.

## 1. Introduction

Marriage is amongst the most important social, cultural and biological aspects of human life that is affected by the changes in communities and can also affect the family and reproductive changes. Marriage and the family formation process changes when the social system is modified or, in other words, adapting to socio-economic conditions. As G. Good asserted, the macro-global processes such as modernization, industrialization and urbanization have changed the traditional structure of families (Abbasi-Shavazi et al., 2009). In the past, due to the influence of kinship networks and strengthening ethnical solidarity, the process of marriage was arranged by more significant others including parents who attempted to choose spouses for their children based mainly on the interests and considerations of the family. However, it seems that the role and autonomy of individual in marriage has increased and the pattern of marriage has changed from a traditional one, planned and selected by parents, to a modern one (Retherford et al., 1996; Choe et al., 2005; Mensch et al., 2005; Moultrie et al., 2012; Ghimire & Axinn, 2013; Abbassi Shovazi & Sadeghi, 2006; Entezari, 2011).

The results of a study by Ogawa in 2003 showed that traditional marriage in Japan between 1955 and 1998 dropped from 63 percent to 7 percent. Likewise, Xiaowei's study (2004) revealed the decrease of traditional pattern of marriage and increase of the romantic relationship and marriage (with furture autonomy in starting and stabilizing the relationship) in different ethnical groups in China since 1949. The results of a survey in Iran in 2005 also showed that 38 percent of marriages took place by the couples’ own selection abbasi (Abbasi-Shavazi & Sadeghi, 2006).

The sociocultural context of marriage and family plays a key role in shaping reproductive behaviors and attitudes abbasi (Abbasi-Shavazi & Asgari Nodoshan, 2005). That is, childbearing, as one of the most important decisions and practices in the life and the main topic of research in the field of demography, should be socio-culturally contextualized (Balbo et al., 2013).

Reproduction, besides the biomedical aspects, also has a socio-cultural form which changes based on various factors in different societies. Opportunities and incentives for childbearing in women are different across populations and subgroups and from society to society. All of these create the differences in the average number of children in women with ethno-culturally diverse background (Weeks, 2010). Morover, human behaviors, including reproductive behavior, is dependent on the social and cultural environment (Morita et al., 2012) and individual and cultural differences that exist in communities lead to different reproductive behaviors (Morgan & Bachrach, 2011).

### 1.1 Literature Review

Review of literature reveals that a number of factors have effects on the reproductive behaviors. These factors are: age, religion, education, mother's employment, maternal age, number of children, age at first pregnancy, access to birth control and contraception methods, healthy lifestyles, family income, type of dating and marriage patterns (traditional, modern and post-modern), ethnicity, having ealier relativeness of couple before marriage (e.g. cousin marriage), women's autonomy, place of birth and urban and rural roots, degree of industrialization, social development, ethnic and cultural beliefs and traditions, the society's regard for the number of family members (McDonald, 2000; Weeks, 2010; Ogawa, 2003; Erfani, 2012; Adhikari, 2010; Dauletova et al., 2012; Morita et al., 2012; Ghimire & Axinn, 2013; Abbassi Shovazi & Asgari Nodoshan, 2005; Abbassi Shovazi & Sadeghi, 2006; Hossein Zadeh et al., 2010).

According to the existing studies, there have been five steps of demographic transition in Iran:

First: decline of fertility in the early 1970s after a long period of high fertility experience in the country;

Second: increased fertility in the period 1976-1980;

Third: the relatively constant progress from 1980 to 1984;

Fourth: slow decline in fertility from 1985 to 1988;

Fifth: the dramatic decline in fertility from 1989 onwards (Abbasi-Shavazi et al., 2009).

Careful study of the document of the first national development program in Iran reveals that, the objective of the family planning program in the late 1980s was to reduce the total fertility rate of 2.3 children per mother by 2010 (Abbasi-Shavazi et al., 2003; Hosseini & Begi, 2012; Abbasi-Shavazi et al., 2004; Abbasi-Shavazi & McDonald, 2006; Hosseini, 2012; Mirzaie, 2005; Mohammad et al., 2002). Indeed, this objective for the family planning program was achieved a decade earlier, so that in 2009, Iran was very close to the replacement level fertility, namely 1.8 children per woman (Salehi-Isfahani et al., 2010).

Since 2009, in which the revision of the official population policy agenda was suggested, two opposite approaches, in connection with Iran's population policy have been discussed: The first approach belongs to the advocates of increase in birth rate and the second approach belongs to the advocates of the management window of opportunity and family planning. The first group believes that in future, the country will suffer from the aging population and decreased workforce. The second group called for a comprehensive population policy for the management window of opportunity and sustaining natural family planning in order to prevent unwanted pregnancy and induced abortion and, finally, to become ready to face the inevitable consequences of the transition in age structure and aging population (Hosseini & Begi, 2012; Hosseini, 2012).

The experience of fertility transition in Iran indicates that although family planning programs are effective in rapidly fertility decrease and its continuation from the second half of the 1980s onwards, the changes in people's reproductive behaviors are influenced by other factors. In fact, the process of fertility transition in Iran is considered proportionate to the changes occurred in various economic, social and some traditional family aspects, leading to the related changes in patterns of marriage and, ultimately, changes in behavior, ideals and aspirations of the people for childbearing. The advocates of the management window of opportunity believe thatany practical application in the field of fertility should be based on economic, social, cultural and demographic realities of Iranian society, and the causes of low fertility should be examined before implementing any program (Calwell et al., 2002; Hosseini, 2012; Hosseini & Begi, 2012; Abbasi-Shavazi & Hosseini, 2009).

As mentioned in previous studies, one of the factors affecting fertility has been the changes in marriage patterns. Researchers believe that changes in the pattern of mate selection from traditional to modern, and the increased role of women in family decisions that increased decision-making power has reduced their childbearing (Abbassi Shovazi & Asgari Nodoshan, 2005). In another study, women who had registered marriage (traditional) were compared to women with civil marriage; they tend to have more children (Akinyoade, 2007; Dauletova et al., 2012). The results of Rashid's study (Rashid, 2006) vary from the previous studies which have shown that women who have had a love marriage want to have children, and more children right after their marriage, for the duration and strength of this relationship.

Since the pattern of mate selection may affect reproductive behaviors and reproductive behaviors have an important role in sexual and reproductive health and women's health and also Preserving women's health is not only a basic human right, but it is also essential for the health of all nations and Women's health affects long-term health of theirs, their family members, and community (Baheiraei et al., 2014), knowing reproductive behaviors of women in modern and traditional mate selection patterns is considered very important in health, demographic, economic, political and educational strategic planning. On the other hand, as the reproductive behaviors are influenced by culture and vary from society to society, studying the behaviours and the factors influencing such behaviours in each city and province of the country in order to adopt appropriate health policies seems necessary.

The city of Babol was chosen as the researcher was familiar with the language and the culture of its people, and this familiarity with the dialect of Babol city made it possible to access real data collection; on the other hand, until the start of the study, no study has been done on the reproductive behaviors of women in mate selection patterns. Also, since the city of Babol is geographically located at the center of Mazandaran province, it can be culturally representative of the whole culture of people in this province and the results can be generalized to this province as a whole. Hence, the present investigation studied the childbearing women in reproductive age, according to their type of mate selection pattern.

## 2. Methods

### 2.1 Research Design

This study began in April 2013 and was completed in January 2014. In this cross-sectional study, after getting a letter from the University Ethics Committee with the number of: 92-130-1297, a questionnaire was used to examine “reproductive behaviors of women” in the north of Iran. The questionnaire was developed using an existing questionnaire, namely “the fertility transition questionnaire in Iran,” originally developed by M. J. Abbasi -Shavazi (Abbasi-Shavazi et al., 2009). Then, a panel of experts comprising professionals with diverse academic backgrounds was selected to identify items relevant to the marriage patterns and reproductive behaviors and to improve and perfect the questionnaire. The final questionnaire was done based on the design and evaluation of psychometric study. The psychometric properties assessment of instruments includes the determination of quantitative and qualitative formal validity and the determination of quantitative and qualitative content validity Duval (Duval et al., 2006). For the qualitative content validity, 15 experts suggested to add or remove items, observe proper grammar and use proper words, and for the quantitative content validity, two ratios of content validity ratio (CVR) and the content validity index (CVI) were used. To check the reliability, the questionnaire was completed in two stages, within two weeks by the 20 married women of reproductive age, and the scores obtained in the test and re-test were compared with each other by the Spearman correlation coefficients. Finally, the questionnaire was used to obtain a sample. Required sample size was determined using data from a pilot study with 85 subjects.

### 2.2 Sampling and Procedure

The samples were 880 married women between 15-49 years old, lived in rural and urban areas in four geographic areas of six districts of Babol city using a multi-stage cluster sampling method. So the first cluster was designated (villages or towns) in these areas and the number of households in each clusterwas divided into the total number of households in four regions; the resulting number was multiplied by the total number of samples (880). After determining the number of samples in each village and town, interviewers attended in the clinics and received the informed consent from eligible clients and questionnaires were completed. Inclusion criteria for participating in this study were being married women of reproductive age (15-49 years), having at least one child and with no diseases or deficiencies such as the primary and secondary infertility, a kind of disease that can cause interference in their fertility, severe mental disability and psychiatric disorders which may cause inability to respond to the questionnaire. The exclusion criteria of this study was the lack of response to the maximum 10% of the questions. Subjects were free to refuse to fill out the questionnaire at any stage of the proceeding. After completing the questionnaires, data were analyzed using IBM SPSS software (version 21).

### 2.3 Data Analysis

To manage and analyze data, a number of descriptive and inferential methods of statistics including ANOVA, Chi-square, Kruskal-Wallis and Factorial Analysis of variance were applied.

## 3. Results

In this study, total information including the socio-demographic characterstics was collected from about 880 married women in the reproductive age (15-49 years) from the city of Babol in the north of Iran (Table 1).

**Table 1:** Socio-demographic characterstics of married women (15-49 years) in Babol, in the north of Iran

Socio-demographic profile	Frequency	%	Socio-demographic profile	Frequency	%
**Age**			**The economic situation**			
15-25	111	12.6	Low	184	20.8
26-35	371	42.2	Medium	534	60.7
36-45	293	33.3	High	160	18.2
46-49	105	11.9			
**Level of education**			**Current employment status**		
Illiterate	38	4.3	Unemployed	625	71.022
Primary school	149	16.9	Laborer	37	4.2
Guidance school	152	17.3	Employee	103	11.7
High School/Diploma	323	36.7	Freelance	91	10.34
Bachelor and higher	221	25.1	Student	24	2.727
**Place of Birth**			**Current place of living**		
City	349	39.7	City	485	55.1
Rural	531	60.3	Rural	395	44.9
**Employment status before the first pregnancy**			**Age of marriage**		
Unemployed	677	76.9	<15	114	13.0
Laborer	29	3.3	15-19	424	48.2
Employee	85	9.7	20-25	275	31.2
Freelance	56	6.4	26-30	55	6.2
Student	33	3.8	>30	12	1.3
**Family connections before marriage**			**Living with family (first 2 years of marriage)**		
Relative	153	17.4	With at least one of my own family members	22	2.5
Unrelated (with pre-familiarity)	225	25.6	With at least one of my husband's family members	409	46.5
Unrelated(without pre-familiarity)	502	57.0	None	445	50.6

Following a review by the research team and modern and traditional definitions of marriage from various works (Xiaohe & Whyte, 1990; Ghimire et al., 2006), marriage patterns classified into 8 clusters or categories based on the initial classification of the panel of experts, and integrated with 3 traditional, modern and integrated patterns based on three items (selecting mate by parents, romantic relationship and relations before marriage and satisfaction in the time of marriage). According to the results of this study, the integrated marriage patterns had the highest frequency (77.2%) and the pattern of modern marriage (10.9%) had the lowest frequency with very little difference in the pattern of traditional marriage (7.11%) (Table 2).

**Table 2:** Marriage patterns of the participated married women (15-49 years) in Babol, Iran (first division)

Pattern of Marriage	Frequency	%
**1. Traditional**	103	11.7
• I did not know my husband before marriage, we met each other through matchmaking and I was not really satisfied and I was somehow forced to marry him.	79	9
• I knew my husband before marriage and through matchmaking and somehow with no satisfaction I was really forced to marry him.	21	2.4
• Our marriage was a sort of arranged marriage (marriage with parental consent like uncle and a cousin marriage, marriage agreement, etc.).	3	0.3
**2. Integrated**	**679**	**77.2**
• I knew my husband before marriage and I was married through matchmaking and and with the approval and satisfaction of myself and my family.	212	24.1
• We did not know each other before marriage. I met him through matchmaking and we married with my consent and my family's approval.	467	53.1
**3. Modern**	**98**	**11.1**
• We were intrigued and wanted each other before marriage and married with our family's approval.	72	8.2
• We were intrigued and wanted each other before marriage and married despite the objections of our families.	24	2.7
• Even though we were friends before our marriage, I was not going to marry him and I was actually kind forced to marry him.	2	0.2
**4. Please note other patterns …**	**0**	**0**

Total	880	100

In this study, a number of bivariate statistical methods including ANOVA, Chi-square test and Kruskal-Wallis test was used to examine the difference among the three patterns of marriage in terms of socio-demographic characteristics. Plus, the Factorial Analysis of Variance was applied to examine the main effect of marriage patterns on the average number of children in married women. The results of these tests showed that these three patterns of marriage are not statistically significant in terms of socio-demographic variables such as education, place of birth, current place of life, employment status of woman before the first pregnancy, current employment status, independent living place during the first two years of marriage and marriage age. However, age of samples in modern marriage patterns (32.33±84.8) was significantly lower than traditional marriage patterns (23.36±84.8) (p <0.05). Morover, there is no statistically significant difference among the three groups of marriage pattern in the number of gravid, pregnancy or no current pregnancy, experiencing child mortality, the number of sons and daughters, experiencing abortion and unwanted pregnancy.

Difference in the number of children among the three patterns of marriage, as one of the reproductive behaviors was the main aim of this study. One-way ANOVA test revealed that there are no statistically significant differences between three patterns of marriage in the number of children. To remove the effect of demographic variables on child number from marriage patterns, Factorial Analysis of Variance was used. However, this test showed no significant deference in marriage patterns on child number (Table 3).

**Table 3:** The main effect of the marriage patterns on the average number of children in Bobol, Iran

Variables	Mean Square	F	P-value
Age	53.16	157.59	0.000
Education	1.21	3.59	0.058
Place of birth	0.84	2.51	0.11
Residence	0.42	1.26	0.26
Job before pregnancy	0.011	0.03	0.85
Current Job	0.69	2.06	0.15
Age of husband	0.98	2.92	0.08
Husband's education	0.69	2.05	0.15
Occupation of husband	0.43	1.29	0.25
Relationship with husband	0.21	0.63	0.42
Number of marriages	0.88	2.61	0.1
Age at current marriage	0.02	0.05	0.81
Living with relatives in the first two years of marriage			
	0.61	1.81	0.17
Socio-Economic Status (SES)	0.09	0.26	0.6
The Marriage pattern	0.34	1.01	0.36

## 4. Disscussion

In this study, the role of marriage pattern on the most important reproductive behaviors like the number of children was examined. Researchers believe that reproduction varies according to the pattern of marriage and this difference is mainly created by the characteristics of socioeconomic diversity which is associated with the differences in the type of mate selection (Ogunjuyigbe & Adeyemi, 2003).

Nowadays, it seems that the role of families in marriage of their offspring is declining and people's attitudes are changing in this regard. Social changes have rapidly removed arranged marriages by parents’ selection, which also occurred in Asia, Africa and Latin America nearly three decades ago. It seems that the change in the type of marriage has played an important role in childbearing (Ghimire & Axinn, 2013).

Women whose their husbands are chosen by families or relatives marry earlier, have children earlier, and have higher fertility levels as they are more prone to fertility from the marriage until menopause. In this type of marriage, girls are more likely to drop out of school due to early marriage. Thus, lower education levels, which reduce the awareness of family planning methods, may be another factor in the high fertility levels. The age difference between spouses in traditional marriages is usually higher, which results in less interaction between couples regarding a suitable and more desirable number of children, as well as the use of contraceptives, thereby increasing the level of fertility. In contrast, women who participate in choosing their husbands typically have a higher education level (Smith et al., 2009; Ogunjuyigbe & Adeyemi, 2003) and, because of greater awareness and increased use of contraceptives, increased decision-making power in the household, especially in the case of fertility, providing job opportunities and greater interaction between couples before and after marriage (because of lowerage difference), have lower fertility levels (Ogunjuyigbe & Adeyemi, 2003).

In the present study, the pattern of marriage of women of childbearing age had no effect on the average number of children, which is different from the results of some studies which showed fertility is higher in traditional pattern of marriage than in modern marriages (Feyisetan & Bankole, 1991; Ghimire & Axinn, 2013) and studies with entirely diverse results (Ogunjuyigbe & Adeyemi, 2003; Rashid, 2006; Akinyoade, 2007; Dauletova et al., 2012) are also different. In a study conducted in the rural areas of Nepal, researchers showed that despite the decline of the family involvement in pattern of marriage and changes in people's attitude towards the pattern of marriage issue, the fertility levels of the women are still high. In this study, the average number of children in the modern marriage was higher than the traditional pattern of marriage and it was perhaps due to the high level of family control on the couples life (Ogunjuyigbe & Adeyemi, 2003).

This study on the main effect of the marriage patterns on reproductive behavior has encountered some limitations as follows: First, many of the macro changes or transitions in socio-cultural, political and economic fieldswhich result in differences in patterns of marriage can also affect the reproductive behaviors in the society (Fricke & Teachman, 1993; Reddy & Modell, 1995; Pallitto & O’Campo, 2004; Abbassi Shovazi & Sadeghi, 2006). Hence, the study of the main effect of marriage patterns on the reproductive behaviors including the number of children requires careful consideration of all socio-cultural, economic, and political underlying factors that may form reproductive behaviors, schemas and attitudes. Second, the marriage pattern cannot be considered independent from the marriage timing. As recognized in the literature, the marriage timing is an important factor in explaining reproductive behavior (Haddad, 2012; Smock & Greenland, 2010; Schuler et al., 2006; Rashid, 2006; Mensch et al., 2005; Choe et al., 2005; Wong & Yeoh, 2003; Ogawa, 2003; Makoto, 2001; Retherford et al., 1996; Bongaarts, 1994; Bankipour Fard et al., 2011).

As a result, marriage timing must be considered at the same time of the investigation on the role of marriage patterns on childbearing. In the present study, these factors were also studied. Due to the differences between the findings of this study and some of the existing studies, it seems that today, there is a convergence in reproductive behaviors due to the marriage selection patterns and different socioeconomic backgrounds. This finding of our study is similar to a previous enquiry by Abbasi *et al* (Abbasi-Shavazi et al., 2004) in Iran. Their observation showed that reproductive behaviors and attitudes in all age groups are similar, and there are no significant differences among different social groups including rural and urban, literate and illiterate women. Likewise, Ogawa (Ogawa, 2003) in a study in Japan did not observe any relationship between the type of marriage (traditional or modern) and interval between marriage and first pregnancy, which is one of the most important variables in women's fertility.

## 5. Conclution

In summary, the evidence in this study indicated that changes in marriage process and the marriage patterns like the transition from traditional to modern or integrated models, cannot make significant changes in the reproductive behaviors in the contemporary Iran. So, according to the new demographic polices of Iran to increase the population, it seems that policy makers should pay more attention to the Macrolevel determinants of fertility such as socio-economic status. We think the convergence in reproductive behaviors there are in other province of Iran too and the researcher should study this phenomen to policy-makers can have proper planning in the field of reproductive behaviors of Iranian people.
